# Phytochemical Analysis and Understanding the Antioxidant and Anticancer Properties of Methanol Extract from *Litsea glutinosa*: In Vitro and In Vivo Studies

**DOI:** 10.3390/molecules27206964

**Published:** 2022-10-17

**Authors:** Shafia Shafiq, Ronok Zahan, Samina Yesmin, Alam Khan, Md. Sabbir Mahmud, Md Abu Reza, Sarah M. Albogami, Mohammed Alorabi, Michel De Waard, Hebatallah M. Saad, Jean-Marc Sabatier, Tarannum Naz, Gaber El-Saber Batiha

**Affiliations:** 1 Department of Pharmacy, University of Rajshahi, Rajshahi 6205, Bangladesh; 2 Biomedical and Toxicological Research Institute (BTRI), BCSIR, Dhaka 1205, Bangladesh; 3 Department of Genetic Engineering and Biotechnology, University of Rajshahi, Rajshahi 6205, Bangladesh; 4 Department of Biotechnology, College of Science, Taif University, P.O. Box 11099, Taif 21944, Saudi Arabia; 5 Smartox Biotechnology, 6 rue des Platanes, 38120 Saint-Egrève, France; 6 L’institut du Thorax, INSERM, CNRS, Université de Nantes, 44007 Nantes, France; 7 LabEx «Ion Channels, Science & Therapeutics», Université de Nice Sophia-Antipolis, 06560 Valbonne, France; 8 Department of Pathology, Faculty of Veterinary Medicine, Matrouh University, Mersa Matruh 51744, Egypt; 9 Institut de Neurophysiopathologie (INP), CNRS UMR 7051, Faculté des Scienes Médicales et Paramédicales, Aix-Marseille Université, 27 Bd Jean Moulin, 13005 Marseille, France; 10 Department of Pharmacy, Varendra University, Rajshahi 6204, Bangladesh; 11 Department of Pharmacology and Therapeutics, Faculty of Veterinary Medicine, Damanhour University, Damanhour 22511, Egypt

**Keywords:** antioxidant, DPPH, anticancer, apoptosis, gas-chromatography–mass-spectrometry, ethnomedicinal plants

## Abstract

*Litsea glutinosa* (*L. glutinosa*) is considered an evidence-based medicinal plant for the treatment of cancer, the leading cause of death worldwide. In our study, the in vitro antioxidant and in vivo anticancer properties of an essential ethno-medicinal plant, *L. glutinosa*, were examined using non-toxic doses and a phytochemical analysis was executed using gas-chromatography–mass-spectrometry. The in vitro antioxidant study of the *L. glutinosa* methanolic extract (LGBME) revealed a concentration-dependent antioxidant property. The bark extract showed promising antioxidant effects in the 2,2-diphenyl-1-picryl-hydrazyl (DPPH) assay. The strongest antioxidant activity was demonstrated at the maximum concentration (50 µg/mL). The IC_50_ values of the LGBME and BHT were 5.51 and 5.01 µg/mL, respectively. At the same concentration, the total antioxidant capacity of the LGBME was 0.161 µg/mL and the ferric reducing antioxidant power assay result of the LGBME was 1.783 µg/mL. In the cytotoxicity study, the LD_50_ of the LGBME and gallic acid were 24.93 µg/mL and 7.23 µg/mL, respectively. In the in vivo anticancer-activity studies, the LGBME, particularly at a dose of 150 mg/kg/bw, showed significant cell-growth inhibition, decreased tumor weight, increased mean survival rate, and upregulated the reduced hematological parameters in EAC (Ehrlich’s ascites carcinoma)-induced Swiss albino mice. The highest cell-growth inhibition, 85.76%, was observed with the dose of 150 mg/kg/bw. Furthermore, the upregulation of pro-apoptotic genes (p53, Bax) and the downregulation of anti-apoptotic Bcl-2 were observed. In conclusion, LGBME extract has several bioactive phytoconstituents, which confirms the antioxidant and anticancer properties of *L. glutinosa.*

## 1. Introduction

Cancer is a complex disease with several etiologies, and current medicines have a slew of adverse effects, posing a significant economic burden [1]. An estimation of cancer incidence, mortality, and prevalence at the national level for 184 countries worldwide in 2012 was conducted by the International Agency for Research on Cancer. It revealed 14.1 million new cancer cases, 8.2 million cancer deaths, and 32.6 million cancer survivors (within five years of diagnosis) [2]. These values may increase to 26 million new cancer cases and 17 million cancer deaths worldwide per year by 2030 [3]. Today, despite significant efforts, cancer remains lethal on a global scale. Additionally, despite their high development costs, novel synthetic chemotherapeutic agents now in clinical use have fallen short of expectations during the last decade. As a result, discovering novel, effective, and affordable anticancer drugs is a constant necessity [4]. The efficiency of phytochemical combinations in cancer therapy may be attributed to their capacity to activate many signaling pathways that induce cell death, restrict cell proliferation and invasion, sensitize malignant cells, and enhance the immune system [5,6]. At present, nearly 60% of the drugs used for cancer treatment are of natural origin [7,8]. The plant kingdom is considered the most significant source of drugs with anticancer activities, including vinca alkaloids, diterpenes, camptothecin alkaloids, and *Podophyllum lignans*. Furthermore, 16 new compounds from plant sources are undergoing clinical trials, among which thirteen are in phase I or II, and three are in phase III. Compounds such as flavopiridol, found in the Indian tree *Dysoxylum binectariferum*, and meisoindigo, also found in the Chinese plant *Indigofera tinctoria*, exhibited anticancer effects with minimal toxicity compared to conventional drugs [9]. The plant *Litsea glutinosa* (*L. glutinosa*) (Lour.) C. B. Rob. (*Lauraceae*) [10] has been selected to evaluate its antioxidant, cytotoxicity, anticancer, and apoptotic properties. It is a semi-evergreen tree species traditionally used for treating various human ailments and diseases. Almost all parts of the plant are used traditionally, but the bark has high commercial value. Popularly known as ‘Menda’, it grows wild in the forest of Chittagong and in the Sylhet districts in Bangladesh [11]. Locally, it is known as Kukurchita, Menda, Pislamenda, Kajlipata, Fotekka, or Roton. All the parts of *L. glutinosa* are utilized in traditional medicine or personal-care items, such as natural cleaning products, as the leaves contain natural saponins [12]. The oil extracted from the stem bark is used as a mild astringent and demulcent in treating diarrhea and dysentery to relieve pain. It is also used to boost sexual desire and treating sore eyes, aches, skin infections, gout, and wounds. Furthermore, it is used to produce a soothing effect on the body [13,14] and to treat cancerous tumors [15].

Previous in vitro cytotoxic-activity studies of the ethyl-acetate extract of *L. glutinosa* (leaves and twigs) against HeLa (human carcinoma of the cervix) demonstrated promising cytotoxic activity [10,16]. The dichloromethane (DCM) fraction of *L. glutinosa* also showed significant anti-proliferative activity against some cancer-cell lines in MTS cell-proliferation assays with different IC_50_ values for different cell lines, such as primary neonatal human epidermal keratinocytes (nHEK), HT29 (adenocarcinoma), and Jurkat E6-1 (clone) [17]. Isolated compounds (pallidine and predicentrine) showed moderate cytotoxicity against the cancer cell lines KB, HepG2, Lu, and MCF7 [18]. *Litsea rubescens* and *Litsea pedunculata,* the two other plants from the genus Litsea, contain two novel flavonoids with chalcone skeleton, and seven known flavonoids also displayed potent cytotoxic activities against myeloid leukemia (HL-60) and epidermoid carcinoma (A431) cell lines in comparison to the drug cisplatin (DDP) [19]. Boldine, which is one of the significant *L. glutinosa* analogs, showed anticancer activity against 3K51, 4J6G, 4KGG, 3 MHD, and 4MSV, and molecular docking revealed that 4MSV is the best protein with which boldine interacts efficiently [20]. There is no anticancer-activity study of *L. glutinosa* using in vivo models in the published literature. Therefore, considering the promising anticancer activity of the plants from the *Litsea* genus and different species, including *L. glutinosa*, and the lack of previous studies on animal models, the present study was designed to evaluate the antioxidant and anticancer activities (in vivo) of *L. glutinosa* bark (LGBME) and to examine the expression levels of three distinct apoptotic genes (P53, Bax, and Bcl-2) to confirm its apoptotic effect.

## 2. Results

### 2.1. Phytochemical Components of LGBME

The LGBME was found to have a large amount of phenolics 126.34 mg of GAE/g of dried extract. It also contained a good amount of flavonoids, 297.93 mg of CAE/g of dried extract (Table 1).

### 2.2. Chemical Compounds of LGBME

**Figure 1:**
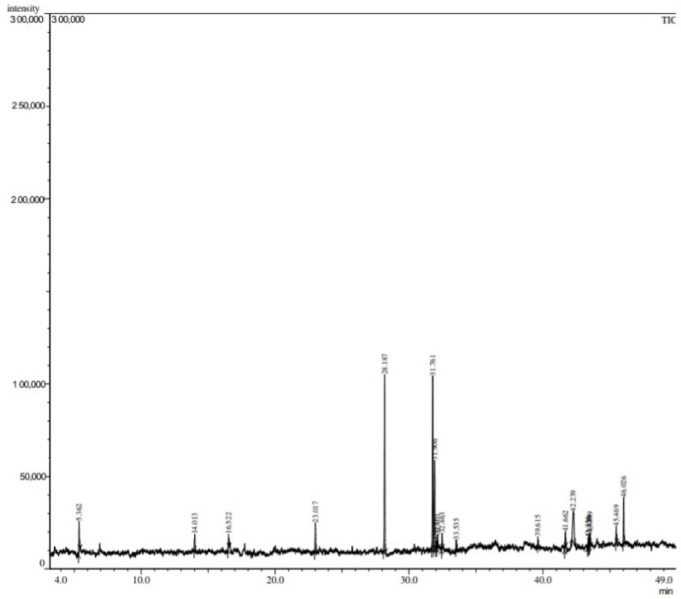
GC–MS analysis of LGBME.

The GC-MS analysis of the LGBME suggested identifying 19 compounds from different chemical families. The suggested compounds, with their retention time and peak area (%), are presented in Table 2 and Figure 1.

### 2.3. In Vitro Antioxidant Activity of LGBME

**Figure 2:**
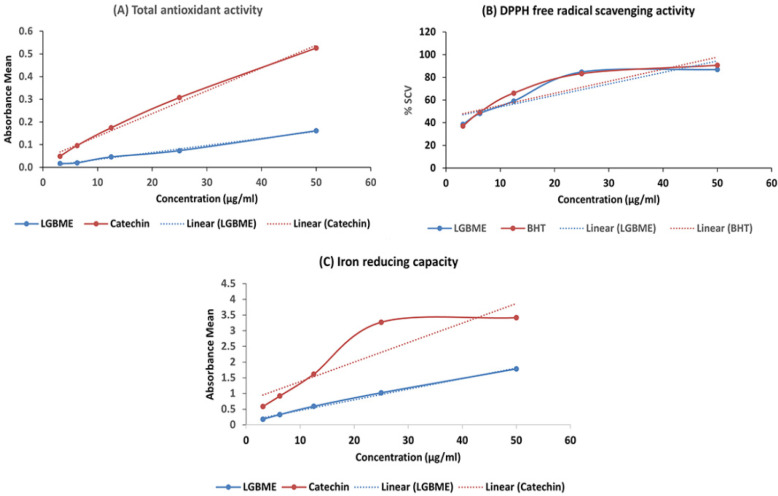
Antioxidant activity of LGBME. (**A**) Total antioxidant activity of LGBME and catechin; (**B**) DPPH free-radical-scavenging activity of LGBME and BHT; (**C**) iron-reducing capacity of LGBME and catechin.

The dose-dependent antioxidant activity of the LGBME was observed. The sample showed intense radical-scavenging activity against DPPH (2,2-diphenyl-1-picryl-hydrazyl) in a concentration-dependent manner (IC_50_ value 5.51) compared with the standard BHT (IC_50_ value 5.01) (Figure 2B). The mean absorbances of the LGBME and standard catechin at concentrations of 50 µg/mL were 0.161 ± 0.016 and 0.526 ± 0.001, respectively, measured at 695 nm (Figure 2A) in a total-antioxidant capacity (TAC) Assay. In addition, the LGBME and catechin demonstrated promising antioxidant activity in a ferric reducing antioxidant power (FRAP) assay in a concentration-dependent manner, when taken at 700 nm. The absorbances of the LGBME and standard catechin at the concentrations mentioned above were 1.783 ± 0.008 and 3.417 ± 0.005, respectively (Figure 2C).

### 2.4. Cytotoxic-Activity Study of LGBME

A brine-shrimp lethality bioassay was used to assess the LGBME’s cytotoxic activity against nauplii. The LC_50_ value was 24.93 μg/mL, while that of the gallic acid was 7.23 μg/mL (Table 3).

### 2.5. Acute-Toxicity Study of LGBME

The main purpose of the acute-toxicity study was to establish safe doses for the experiment. The activity of the investigational model was observed for the first 3 h and, later, at intervals of 4 h throughout the next 24 h. The foodstuff ingestion exhibited no significant alteration amongst the groups that were monitored. In this study, the mortality rate was null at 250, 500, 1000, and 1500 mg/kg/bw. This indicated that the LD_50_ was much higher than 1500 mg/kg/bw.

### 2.6. Anticancer Activity of LGBME

**Figure 3:**
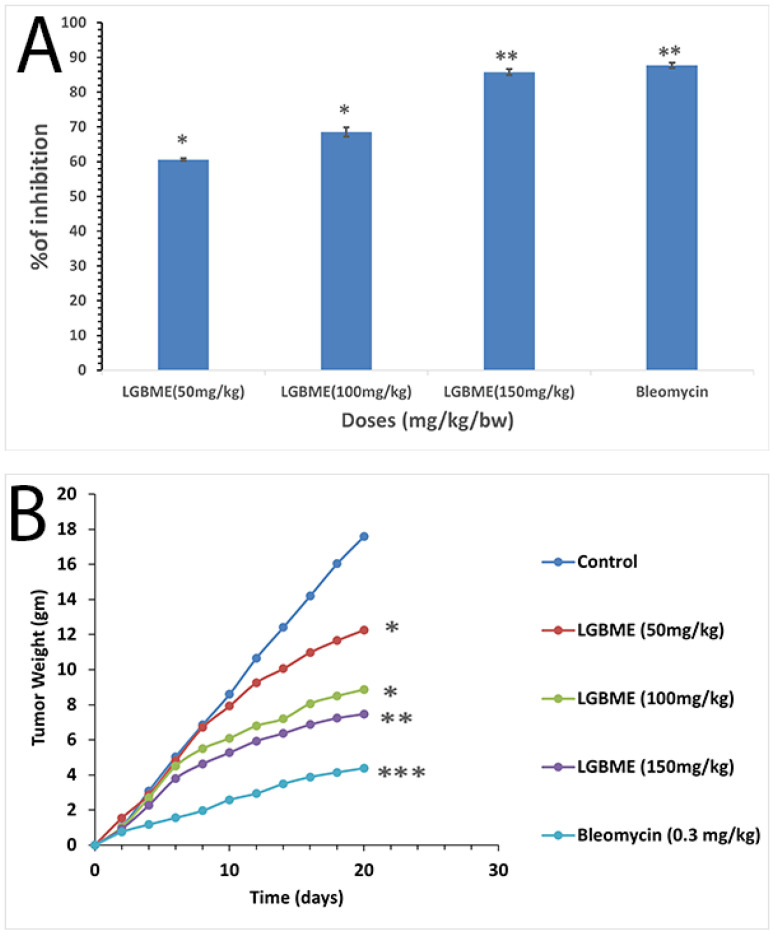
Anticancer activity of LGBME. (**A**) Effects of LGBME on EAC cell-growth inhibition; (**B**) effects of LGBME on tumor weight of EAC-bearing mice; results are shown as mean ± SD (*n* = 6), where significant values are set as * *p* < 0.05, ** *p* < 0.01, and *** *p* < 0.001 for treated (EAC + LGBME) mice compared with EAC-bearing control mice.

The viability of the EAC cells decreased considerably when they were treated with various doses of LGBME in a concentration-dependent manner (Figure 3A). Significant EAC cell-growth inhibition (85.76%) with LGBME was observed at the dose of 150 mg/kg.bw/day (i.p.), at doses 50 and 100 mg/kg.bw/day; the values were 60.60% and 68.53%, respectively (*p* < 0.05), and significant cell-growth inhibition (87.70%) resulted from the use of the standard drug, bleomycin, at 0.3 mg/kg.bw/day, when compared with the control (*p* < 0.01).

Increased mean survival time (MST) and life span were observed in a dose-dependent manner during the treatment with the LGBME. The MST of the control-mice group was 21.67 ± 0.58, and that of the treatment group was 37.5 ± 1.29 days (*p* < 0.05) at the dose of 150 mg/kg/day (Table 4) and at doses 50 and 100 mg/kg.bw/day; the values were 29.67 ± 2.65 and 36.33 ± 1.53 days, respectively (*p* < 0.05). The MST of the standard drug, bleomycin, at 0.3 mg/kg.bw/day, was 40.0 ± 2.08 days (*p* < 0.001). Significantly increased life span resulted from the treatment with the LGBME dose of 150 mg/kg/day, which was 73.05% compared to the control group; for the standard bleomycin, the value was 82.08%.

The effects of the LGBME and bleomycin on the EAC-cell-inoculated mice resulted in dose-dependent tumor-growth inhibition (Figure 3A). The tumor weights of the control group were elevated by 17.6 g, and in the case of the mice treated with the LGBME at doses of 50, 100, and 150 mg/kg.bw/day, the values were 12.26, 8.86, and 7.47 g, respectively (*p* < 0.05). The results are shown in Figure 3B. All the groups were compared against the control group using daily weight changes after 20 days of treatment.

The hematological parameters were remarkably decreased in the EAC-cell-bearing control-mouse group compared with the normal-mouse group. However, with the continuation of the LGBME treatment, these parameters (hemoglobin, RBC, and WBC) were reversed towards normal values (Table 5).

### 2.7. Effects of LGBME on Apoptotic Genes

**Figure 4:**
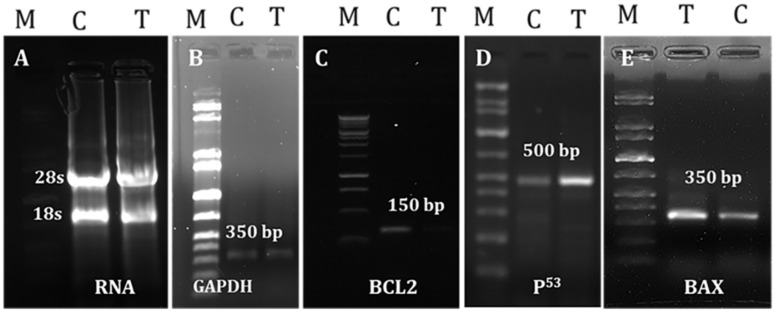
Study of the mRNA-expression pattern of apoptotic-related genes through RT-PCR. C, T, and M refer to EAC-cell-bearing control mice (untreated), LGBME-treated mice, and molecular marker, respectively. The band length is indicated as bp. (**A**) Isolated RNA from both control and treated EAC cells in 1% agarose-gel electrophoresis; (**B**) expression of housekeeping gene, GAPDH; (**C**) expression of the anti-apoptotic gene, Bcl-2; (**D**,**E**) expression of the pro-apoptotic genes, p53 and Bax.

GAPDH was consistently expressed in both the control and the LGBME-treated groups. The p53 was found to be expressed in the LGBME-treated EAC cells at the dose of 150 mg/kg.bw/day; thereby, the expression of the pro-apoptotic gene Bax was increased in the treated cells compared with the control cells. By contrast, the expression of the anti-apoptotic gene Bcl-2 was decreased in the treated cells compared to the controls. A molecular study of the apoptosis-related genes revealed that the upregulation of p53 and Bax and the downregulation of anti-apoptotic Bcl-2 might be responsible for EAC-cellgrowth inhibition by inducing apoptosis (Figure 4).

### 2.8. Histopathological Findings of Liver and Kidney Tissues

**Figure 5:**
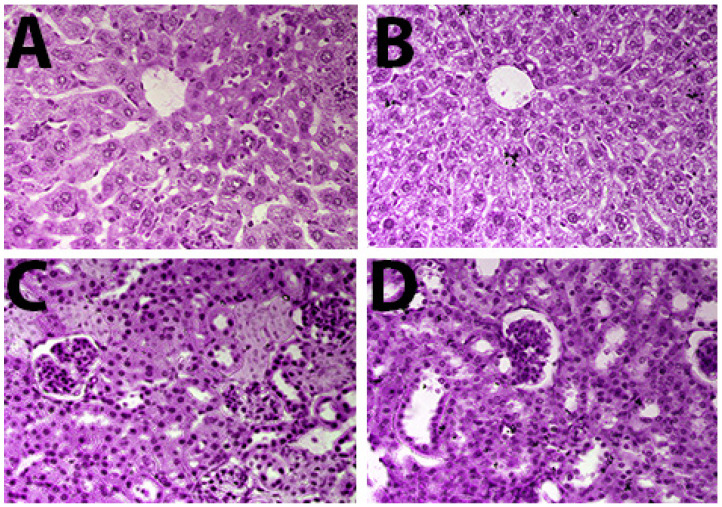
Photomicrographs of H&E-stained liver and kidney sections from *Swiss albino* mice: (**A**) EAC control liver; (**B**) LGBME-treated liver; (**C**) EAC control kidney; (**D**) LGBME-treated kidney. All photographs magnified 10 × 40x.

A histopathologic examination revealed remarkable differences in the liver and kidney cytoarchitectures on the sixth day of cell inoculation in the LGBME-treated mice compared to the control-mouse group (Figure 5A,B). In the liver sections of the control mice, we found ballooning degeneration, hepatocellular necrosis, higher fatty change, several pyknotic cells, degenerate nuclei, hyperplasia of the Kupffer cells surrounding the central vein, and irregularly shaped central veins, whereas the LGBME (150 mg/kg.bw/day)-treated mice showed restoration of their hepatic architecture with regular-shaped central veins, and hepatocellular necrosis was not found. In addition, pyknotic cells and the Kupffer cells surrounding the central vein were found in fewer quantities than in the control group. A microscopic examination of the kidney sections from both the control and the treated mouse groups showed no significant disturbance in the kidney cytoarchitectures at the same dose (Figure 5C,D).

## 3. Discussion

Traditional herbal medicines have well-recognized uses as complementary and alternative medicines (CAMs) to manage, prevent, or cure a wide range of human diseases, including cancer [21]. Phytochemicals, such as phenolics and flavonoids, are the most copious antioxidants and plant species containing a significant amount of phenolics and flavonoids have been found to be therapeutically efficient in treating cancers [22]. According to previous studies, polyphenolic compounds have been proven to have cytotoxic and apoptotic effects on various cell lines while protecting normal cells from detrimental effects [23,24]. In our study, the LGBME contained phenolics and flavonoids (Table 1). A previous study suggested that elevated polyphenol (phenolic and flavonoid) content strengthens antioxidant activity [25].

Reactive oxygen species play crucial roles in cancer initiation, and progression and elevated levels were observed in malignant cells compared with their regular counterparts [26,27]. Several anti-oxidative compounds have been shown to protect against experimental carcinogenesis and induce the apoptosis of cancer cells [28]. In this work, LGBME showed intense antioxidant activity in the performed assays (Figure 2). Furthermore, the strong scavenging capacity of the LGBME with IC_50_ (5.51 µg/mL) against the standard BHT was found in the DPPH-scavenging-activity assay, supporting the findings of a previous study [14].

The GC–MS analysis of the LGBME also supported the current findings. The identified compounds of bark extract (LGBME)-caryophyllene and 9-Octadecenoic acid (Z)-methyl ester (oleic acid methyl ester) are known for their antioxidant properties [29,30,31,32,33].

For toxicity determination, brine-shrimp nauplii cytotoxicity assay is an old but standardized and effective method. The LD_50_ value of the LGBME was 24.93 ± 1.04 μg/mL (Table 3). From this result, we can say that the plant extract has toxic effects.

For judging plant extracts as potent anticancer agents, the growth inhibition of viable EAC cells, reduction in tumor burden, increase in survival time, and restoration of hematological parameters towards normal levels are considered criteria that need to be fulfilled [21]. These all were satisfied by the LGBME when compared with the results obtained by a simultaneous experiment with a clinically recognized anticancer drug bleomycin, at a dose of 0.3 mg/kg.bw/day) (Figure 3 and Table 4 and Table 5). In our experiment, the viable cell count of the EAC cells was decreased to 85.76 % at the highest dose (150 mg/kg.bw/day) of the LGBME. Furthermore, a significant percentage of increased life span (73.05%), high survival time (37.5 days), and remarkably reduced tumor burden were observed compared with the control group at the same dose (Table 4), and the obtained results were statistically significant (*p* < 0.05). Additionally, the treatment with LGBME brought the hemoglobin content, RBC, and WBC cell count back to near-normal values (Table 5). Furthermore, the presence of some important bioactive compounds, such as δ-Cadinene, 9-Octadecenoic acid methyl ester, di-n-octyl phthalate, etc., was confirmed by the GC-MS analysis (Table 2 and Figure 1). Among these, δ-Cadinene showed promising apoptotic activity through the growth retardation of ovarian cancer (OVCAR-3) cells via caspase-dependent apoptosis and cell-cycle arrest [34], and the di-n-octyl phthalate showed antitumor activity via the inhibition of tumor-necrosis-factor production [35]. Moreover, a medicinal practitioner of Chittagong hill tracts uses *L. glutinosa* to treat cancer and cancerous tumors. Therefore, the present anticancer activity studies on *L. glutinosa* support its ethnomedicinal use in treating cancer and cancerous tumors [15].

According to the acute toxicity study, the LGBME at the dose of 150 mg/kg.bw/day was within the safety margin, as LD_50_ should be more than 1500 mg/kg. This was supported by the histopathological examination of the liver and kidney tissues. No toxic changes in the cytoarchitectures of the examined tissues were observed when compared to the control-group mice. Moreover, it was found that 150 mg/kg.bw/day showed the most promising anticancer activity against EAC cells of the three doses. Therefore, the dose 150 mg/kg.bw/day was chosen to evaluate the apoptotic efficiency. The mechanisms of apoptosis through which the LGBME inhibited cell growth and induced apoptosis in the EAC cells were investigated by analyzing the amplification pattern of some well-known apoptosis-related genes. EAC is an undifferentiated ascites carcinoma cell in mice. These cells produce native p53, Bax, and Bcl-2 proteins, as shown by previous studies. In these studies, the downregulation of Bcl-2 protein and the up-regulation of Bax and p53 proteins, induced by Poly-L-Lysine, was also confirmed in EAC cells via Western blot [36].

Proliferation disorder and apoptosis obstacle are well-known characteristics of cancer [37]. Recent studies suggested decreased physiological cell death, i.e., apoptosis, may be responsible for malignancy rather than elevated levels of cell proliferation. As a result, the induction of apoptosis may serve as a new tactic for innovative mechanism-based drug discovery [38,39]. The most studied apoptotic proteins are p53, Bax, and Bcl-2 proteins. They act through the activities of their pro-apoptotic and anti-apoptotic products, thereby serving as indicators of the success or failure of apoptosis. Several studies revealed the high expression of the tumor-suppressor protein p53, followed by the accumulation of the pro-apoptotic protein Bax, which activated the intrinsic pathway of apoptosis [40,41].

Additionally, Bax is considered an early event that sensitizes cells to undergo apoptosis. Some previous studies proposed that Bax upregulation alone can commit a cell to apoptosis [42]. In addition to the action of Bax and the cell cycle, p53 may also suppress Bcl-2; hence, its anti-apoptosis function is impeded [43]. The equilibrium between the pro-apoptotic and anti-apoptotic properties of the Bcl-2 family plays a vital role in actuating cells toward apoptosis [44]. Previous studies have revealed that the upregulation of Bax and the downregulation of Bcl-2 cause susceptibility to the intrinsic pathway of apoptosis [45,46]. In our study, the expression patterns of p53, Bax, and Bcl-2 were studied, and it was found that treatment with LGBME at doses of 150 mg/kg.bw/day induced apoptosis, which was associated with the upregulation of the tumor-suppressor gene p53 and the pro-apoptotic gene Bax and with the downregulation of Bcl-2 expression in EAC cells.

## 4. Materials and Methods

### 4.1. Chemicals and Reagents

In our study, chemicals and reagents used were of analytical grade and purchased from Sigma-Aldrich (St. Louis, MO, USA). In addition, Oligo (dT), dNTPs, and M-MLV reverse transcriptase were collected from Tiangen (Beijing, China).

### 4.2. Preparation of Extract

The fresh and mature stem bark of plant *L. glutinosa* was collected and authenticated from Bangladesh National Herbarium (BNH) during the autumn season (October 2018) in Mirpur, Dhaka, Bangladesh, with the DACB accession number 48,288. After washing and drying (shed), the bark was ground to a coarse powder and preserved until use at 4 °C. Approximately 0.09 kg powder of bark was soaked in 500 mL of methanol solvent to obtain approximately 0.045 kg of crude methanolic extract, indicated as LGBME. The collection of plant material and all the experimental research and field studies on plants complied with the relevant institutional, national, and international guidelines and legislation.

### 4.3. Determination of Total Phenol and Flavonoid Content

The total phenol and flavonoid [47] content of LGBME were determined spectro-photometrically at 760 nm using Folin–Ciocalteu and aluminum chloride reagent. Total phenolic contents were expressed in terms of gallic-acid equivalent, GAE mg of GA/gm of extract, and the standard curve equation was y = 0.0863x + 0.2372, R^2^ = 0.9896. The total flavonoid content was calculated using a calibration curve and the standard curve equation: y = 0.0051x – 0.0906, R^2^= 0.9944). Results were expressed as catechin equivalents per gram of dried sample (mg of CA/gm).

### 4.4. Antioxidant Activity Study of LGBME

#### 4.4.1. DPPH-Free-Radical-Scavenging-Activity Assay

In DPPH (2,2-diphenyl-1-picryl-hydrazyl)-free-radical-scavenging-activity assay, LGBME was tested at 517 nm [47] and the standard curve equation: y = 1.061x + 44.682, R^2^= 0.8082.

The following formula was used to calculate the free-radical-scavenging activity of the sample and standard radical-scavenging rate (%) = [{A_0_—A}/A_0_] × 100. “A_0_” (blank) denotes the absorbance of DPPH blank solution and “A” denotes the final absorbance of the tested sample after 30 min of incubation.

#### 4.4.2. Total-Antioxidant-Activity Assay

The antioxidant activity of the LGBME was evaluated by the phosphor-molybdenum method [47]. Absorbance was taken at 695 nm. Increased absorbance of the reaction mixture indicated an increased total-antioxidant activity. Standard curve equation: y = 0.0027x + 0.0074, R^2^ = 0.9959.

#### 4.4.3. Ferric Reducing Antioxidant Power Assay

The reducing power of LGBME was measured by the ferric reducing method [47]. The amount of Fe^2+^ complex was then monitored by measuring the formation of Perl’s Prussian blue at 700 nm. A concentration-dependent increase in absorbance indicated an increase in reducing capacity. Standard curve equation: y = 0.0338x + 0.1245, R^2^ = 0.9951.

### 4.5. Gas-Chromatography–Mass-Spectrometry (GC–MS) Analysis of LGBME

LGBME was analyzed by GC–MS analysis technique performed by GC (Model CP-3800, Varian, Santa Clara, CA, USA) with MS (model: Varian Saturn-2200) spectrometer equipped with a flame-ionization detector and capillary column with VF-5 ms (30 m × 0.25 mm, 0.25 μm). The instrument was operated using electron-impact-ionization mode under specified conditions (ionization voltage −70 eV, detector temperature 280 °C, and injector temperature 250 °C). Helium, an inert gas, was used as a carrier gas, the flow rate was 1 mL/minute, and the sample injection volume was 1 μL. The initial temperature for the column was 40 °C (1 min), and the final temperature was 310 °C at a gradually increasing rate of 10 °C/minute. Temperature was held constant at 310 °C for 10 min. Chemical compounds were identified by their GC retention time, retention indices relative to n-alkanes, and comparison of their mass spectra with the NIST 2008 (National Institute Standard and Technology 2008) Library data.

### 4.6. Determination of Cytotoxic Activity

The cytotoxicity of LGBME was evaluated using brine-shrimp-lethality bioassay; nauplii were hatched after 48 h in saline water following a published protocol [48]. Various concentrations of LGBME and standard gallic acid (12.5, 25, 50, 100, and 200 μg/mL) were obtained by serial dilution of 1 mg/mL solution. The percentage of mortality was calculated after 24 h of incubation for the concentrations mentioned above, and the LC_50_ values were determined using Probit analysis.

### 4.7. Experimental Animal and Ethical Clearance

*Swiss albino* male mice 4–6 weeks of age, weighing 27 ± 5 g, were taken from the Department of Pharmacy, University of Rajshahi, and used in this study. Mice were housed in a standard room with a 12-h light/dark cycle and controlled temperature and humidity. Animals were also provided with food and water ad libitum. All experiments were performed in accordance with the institutional and national guidelines for laboratory animals, and approved by the Institutional Animal, Medical Ethics, Bio-safety and Bio-security Committee (IAMEBBC) for Experimentations on Animal, Human, Microbes and Living Natural Sources, Institute of Biological Sciences, University of Rajshahi, Bangladesh (license no: 225/320-IAMEBBC/IBSc). The study was performed in compliance with the ARRIVE guidelines (https://nc3rs.org.uk/arrive-guidelines, accessed on 1 July 2020).

#### 4.7.1. Acute Oral-Toxicity Study

Acute oral-toxicity study was conducted as per the reported method to determine LD_50_ [49]. The injected solution was obtained by dissolving LGBME in 2% DMSO in PBS, and intraperitoneal injections were given to *Swiss albino* mice.

#### 4.7.2. In Vivo Anticancer-Activity Study

In vivo anticancer activity of LGBME was determined by measuring the effect of samples on tumor-cell-growth inhibition, body weight, survival time, and hematological parameters of EAC cell-bearing mice.

#### 4.7.3. Experimental Tumor Model and Preparation of Test Samples

EAC (Ehrlich’s ascites carcinoma) cells were used for in vivo anticancer-activity study in our study. First, the initial inoculums of EAC cells were collected from the Indian Institute of Chemical Biology (IICB), Kolkata, India. Next, the EAC cells were cultured and maintained in our laboratory as ascites tumor *Swiss albino* mice by intraperitoneal injection bi-weekly (1 × 10^6^ cells/mouse) taken from a donor mouse with 6–7-day-old ascites tumor.

The plant extract was given through an intraperitoneal injection to three groups of mice (*n* = 6) at three doses (50, 100, and 150 mg/kg.bw/day), and bleomycin at the dose of 0.3 mg/kg.bw/day was used as standard drug. For this treatment, the crude extract was dissolved in 2% DMSO in PBS; therefore, 100 µL of solution contained the required amount for each dose.

#### 4.7.4. Determination of EAC-Cell-Growth Inhibition

In the cell-growth-inhibition study, at day ‘0’, 1 × 10^6^ EAC cells were given to the five groups of *Swiss albino* mice (*n* = 6) through a peritoneal injection [49,50,51]. Treatments were started after 24 h of tumor inoculation and continued for five consecutive days. Group I was considered as the control group, receiving 2% DMSO only. On the other hand, Groups II–IV received LGBME at doses of 50, 100, and 150 mg/kg.bw/day, and Group V was treated with standard bleomycin at a dose of 0.3 mg/kg.bw/day. After five days of treatment, mice were sacrificed to collect the cells. The viable cells were identified and counted with Trypan blue and hemocytometer, respectively, under an inverted microscope (XDS-1R, Optika, South Korea). The percentage of cell-growth inhibition was calculated using the following formula:

% Cell growth inhibition = (1 − Tw/Cw × 100)(1)

where Tw = mean number of tumor cells of treated groups and Cw = mean number of tumor cells of control groups.

For determination of mean survival time (MST) and percentage increase in life span (%ILS) with average tumor weight, experiment was performed according to a published protocol [49]. For judging therapeutic value, 1 × 10^6^ EAC cells/mL were inoculated per mouse in five groups (*n* = 6) on day 0 and after 24 h of tumor inoculation in the peritoneal cavity; treatments continued for 10 days. Daily weight variation up to 20 days was recorded to calculate the tumor growth. In addition, the survival times of both the LGBME-treated and untreated groups were recorded and expressed as mean survival time (MST) in days, and percentage increase in life span (%ILS) was calculated as follows:

Mean survival Time = (day of 1st death + day of last death)/2(2)

and

% ILS = {(Mean survival time of treated group/Mean survival time of control group) − 1} × 100(3)

#### 4.7.5. Determination of Hematological Parameters

Hematological parameters, such as WBC, RBC, and Hb, were tested by a standard protocol [49]. Counting of total WBC and RBC was performed by using a microscope with a hemocytometer. Hematometer was used to evaluate the percentage of hemoglobin (% Hb).

#### 4.7.6. Evaluation of Apoptotic Effectiveness of LGBME

##### RNA Separation and cDNA Preparation

Isolation of RNA was conducted using RNA simple Total RNA Kit (Tiangen, Beijing, China) following the manufacturer’s guidelines. Concentration and purity of RNA were evaluated by nanodrop 2000 spectrophotometer (Thermo Scientific, Waltham, MA, USA); 3 μg of isolated RNA were converted to cDNA, and reaction mixture contained 10 mM oligo dT (2 μL), M-MLV reverse transcriptase (1 μL), 10 mM dNTPs (2 μL), 5 × 1st strand buffer (4 μL), and sufficient quantity of dH_2_O to make 20 μL volume.

##### PCR Amplification of Apoptosis-Related Genes

Briefly, amplification of cDNA was performed by reverse transcriptase-polymerase chain reaction (RT-PCR) using gene-specific oligos [49]. The expression pattern of apoptosis-related genes, such as p53, BAX, and Bcl- 2 was studied by PCR. GAPDH, a housekeeping gene, was used as a control gene. The subsequent specific oligonucleotides (IDT, Singapore) were used.

*GAPDH* upstream-(5’-GTGGAAGGACTCATGACCACAG-3’) and downstream-(5’-CTGGTGCTCAGTGTAGCCCAG-3’) generated a band of 350 bp; p53 upstream-(5’-GCGTCTTAGAGACAGTTGCCT-3’) and downstream-(5’-GGATAGGTCGGCGGTTCATGC-3’) generated a band of 500 bp; Bax upstream-(5’-GGCCCACCAGCTCTGAGCAGA-3’) and downstream-(5’-GCCACGTGGGCGTCCCAAAGT-3’) generated bands of 500 bp and 350 bp; Bcl-2 upstream-(5’-GTGGAGGAGCTCTTCAGGGA-3’) and downstream-(5’-AGGCACCCAGGGTGATGCAA-3’) generated a band of 150 bp. Amplifications were performed in a Gene Atlas 482 (Japan) thermal cycler. Each 10 µL of PCR reaction mixture comprised 0.20 µL of 10 mM dNTPs, 0.50 µL of templates, 0.40 µL each of 5 mM forward and reverse primer, 0.10 µL of polymerase, and 2 µL of 5X DNA polymerase buffer, and were topped up with deionized water. Reaction conditions were initial PCR activation step of 3 min at 95 °C, followed by 35 cycles of 95 °C for 45 sec, 52 °C for 45 sec, and 72 °C for 1.00 min, and a final extension of 72 °C for 10 min. The PCR product of these oligos and GAPDH were electrophoresed in 1% agarose gel with EtBr. Lastly, the picture was captured under a UV-trans illuminator (Protein Simple).

#### 4.7.7. Histopathologic Evaluation

Livers and kidneys were collected from control (2% DMSO) and treated (dose 150 mg/kg.bw/day) mice after five days of treatment [49], immediately fixed in 10% neutral buffered formalin for at least 24 h, processed by paraffine-embedding technique, stained with hematoxylin and eosin [52], and examined under Motic Advanced system microscope (B, series) with the help of Motic J1.0 software on a Macintosh computer for histopathological changes.

#### 4.7.8. Statistical Analysis

All the analyses were performed in triplicates, and data were expressed as mean ± SD (Standard deviation). Statistical analyses were performed by one-way analysis of variance (ANOVA) followed by Dunnett’s T3-test for evaluating the anticancer activity of LGBME using Statistical Package for Social Science (SPSS) statistical software, v16. A *p*-value < 0.05 was considered to be statistically significant. Levels of significance were tested at 5% (* *p*-value < 0.05), 1% (** *p*-value < 0.01), and 0.1% (*** *p*-value < 0.001), respectively.

## 5. Conclusions

Our findings of antioxidant and anticancer activities lend scientific support to the traditional medicinal uses of *L. glutinosa* for cancer treatment. Several antioxidant and anticancer compounds were discovered in *L. glutinosa* in our study. Understanding the mechanism of action of these therapeutically active compounds may justify the use of medicinal plants for the prevention and/or treatment of disease. The further isolation and characterization of bioactive compounds could provide a lead compound for cancer treatment.

## Figures and Tables

**Table 1:** Quantitative phytochemical analysis of LGBME.

Sample	Total Phenolic Content	Total Flavonoid Content
LGBME	126.34 ± 0.79 ^a^	297.93 ± 4.304 ^b^

The results were expressed as mean ± SD (*n* = 3). The values of ^a^ and ^b^ were expressed in terms of GAE (gallic-acid equivalent) and CAE (catechin equivalent) (mg of GAE/gm, CAE/gm of dry extract, respectively).

**Table 2:** Phytoconstituents detected by GC–MS analysis of LGBME.

SN	Phytoconstituents	RT (min)	Area	Height	Peak Area (%)
1.	p-Cresol	5.362	17,591	4248	9.330
2.	Bicyclo (7.2.0)undec-4-ene, 4,11,11- trimethyl-8-methylene-,(1R-(1R*,4Z,9S*))-	14.013	2967	790	1.573
3.	δ-Cadinene or Naphthalene, 1,2,3,5,6,8a- hexahydro-4,7-dimethyl-1-(1-methylethyl)-, (1S-cis)-	16.522	3208	920	1.701
4.	Methyl tetradecanoate	23.018	12,181	3890	6.460
5.	Hexadecanoic acid, methyl ester	28.187	66,349	23,598	35.192
6.	9,12-Octadecadienoic acid, methyl ester, (E,E)-	31.761	27,755	9934	14.721
7.	Oleic acid methyl ester or 9-Octadecenoic acid (Z)-, methyl ester	31.906	15,498	4490	8.220
8.	15-Octadecenoic acid, methyl ester	32.023	689	313	0.365
9.	3,7,11,15-Tetramethyl-2-hexadecen-1-ol	32.100	1693	585	0.897
10.	Methyl stearate	32.462	4960	1855	2.630
11.	Caryophyllene	33.523	2200	715	1.166
12.	Di-n-octyl phthalate	39.616	3119	1127	1.654
13.	Reticuline, 6’-methyl	41.660	6002	1990	3.183
14.	22,23-Dibromostigmasterol acetate	42.243	3277	665	1.738
15.	benzenemethanol,3-(ethyl(2-hydroxyethyl)amino)-.alpha.-methyl-	43.364	1725	456	0.914
16.	13-Docosenamide, (Z)-	43.436	2059	679	1.092
17.	7-Isoquinolinol, 1,2,3,4-tetrahydro-1-[(3-hydroxy-4-methoxyphenyl)methyl]-6-methoxy-2-methyl-, (S)-	43.497	7145	2472	3.789
18.	6,9,10-Trimethoxy-12H-benz(6,7)oxepino(2,3,4-i,j)isoquinoline	45.468	1925	632	1.021
19.	Cyclotrisiloxane, hexaethyl-	46.024	8190	2520	4.344

RT: Retention time.

**Table 3:** Cytotoxic activity of LGBME.

Test Sample	LD_50_ (μg/mL)	95% Confidence Limits (μg/mL)	Regression Equation	χ^2^ Value (Degrees of Freedom-df)
Gallic acid	7.23 ± 0.44	4.05 to 13.02	y =3.117 + 2.213x	0.049 (1)
LGBME	24.93 ± 1.04	16.95 to 36.66	y = 2.237 + 1.977x	0.325 (1)

**Table 4:** Effect of LGBME on MST and %ILS on EAC-cell-bearing mice.

Name of the Group	MST (in Days)	% of ILS
Control (EAC-cell-bearing mice)	21.67 ± 0.58	-
EAC + Bleomycin (0.3 mg/kg.bw/day)	40.0 ± 2.08 ***	82.08
EAC + LGBME (50 mg/kg. bw/day)	29.67 ± 2.65 *	36.92
EAC + LGBME (100 mg/kg.bw/day)	36.33 ± 1.53 *	67.65
EAC + LGBME (150 mg/kg bw/day	37.5 ± 1.29 *	73.05

Data expressed as mean ± SD (*n* = 6) and significant values at *p* < 0.05 (*), *p* < 0.01 (**), and *p* < 0.001 (***) by Dunnett’s T3 test when compared with the control group.

**Table 5:** Effects of LGBME on hematological parameters in EAC-bearing mice.

Name of the Group	Hb (gm/dL)	RBC (Cells/mL)	WBC (Cells/mL)
Normal mice	14.13 ± 0.72	(7.03 ± 0.11) × 10^9^	(11.6 ± 0.36) × 10^6^
Control (EAC-cell-bearing mice)	6.67 ± 0.55	(4.32 ± 0.21) × 10^9^	(26.8 ± 0.72) × 10^6^
EAC + Bleomycin (0.3 mg/kg, bw)	13.20± 0.94 **	(6.30± 0.26) × 10^9^ **	(14.8 ± 0.95) × 10^6^ **
EAC + LGBME (50 mg/kg, bw)	9.73 ± 0.56 *	(5.39 ± 0.14) × 10^9^ *	(21.0 ± 1.00) × 10^6^ *
EAC + LGBME (100 mg/kg, bw)	11.83 ± 0.68 **	(6.05 ± 0.19) × 10^9^ **	(15.8 ± 0.92) × 10^6^ **
EAC + LGBME +(150 mg/kg, bw)	12.68 ± 0.39 **	(6.21 ± 0.45) × 10^9^ *	(14.7 ± 0.46) × 10^6^ ***

Each calculated value represents mean ± SD (*n* = 6), and the significance test was set up at *p* < 0.05 (*), *p* < 0.01 (**), and *p* < 0.001 (***) by Dunnett’s T3 test; RBC, red blood cells; WBC, white blood cells; Hb, hemoglobin.

## Data Availability

Not applicable.
